# Supplementary material for: BRWD3 promotes KDM5 degradation to maintain H3K4 methylation levels
Source: bioRxiv. 2023 Mar 28:2023.03.28.534572. Preprint. [Version 1] doi: 10.1101/2023.03.28.534572 (PMC10081218; doi:10.1101/2023.03.28.534572)
Supplement: Supplement 1 [file media-1.pdf]

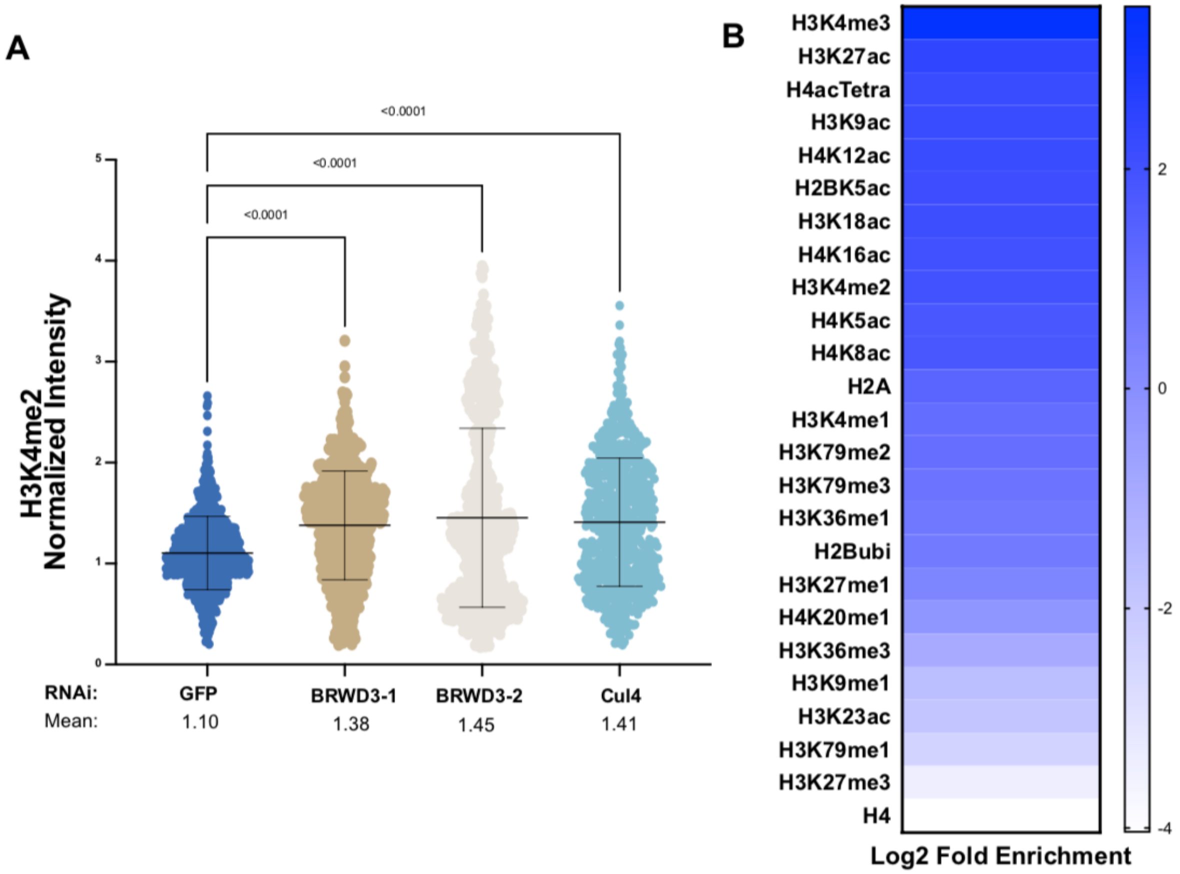

Supplemental Figure 1. BRWD3 affects H3K4 methylation status. (A) Violin plots of quantitative immunofluorescence experiments in Drosophila S2 cells using an anti-H3K4me2 antibodies. Each distribution represents the signal intensities of 1000 randomly selected cells from three biological replicates. ****p < 0.0001 ANOVA one-way analysis with Tukey's multiple comparisons test. (B) Significant enrichment of all published histone modifications within BRWD3 binding sites. Log2-fold enrichment for observed overlap relative to expected overlap for each mark peak set is shown.

A

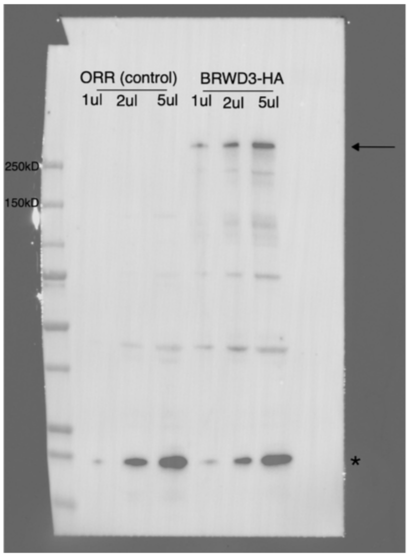

Supplemental Figure 2. Verification of endogenously HA-tagged BRWD3. Western blot samples were prepared from endogenously HA-tagged BRWD3 embryos (0-24h) or ORR negative control embryos (0-24h), probing with an anti-HA antibody. Arrow indicates BRW3-HA (~270 kD). Asterisk indicates non-specific bands recognized by anti-HA antibody (both in ORR control and BRWD3-HA).

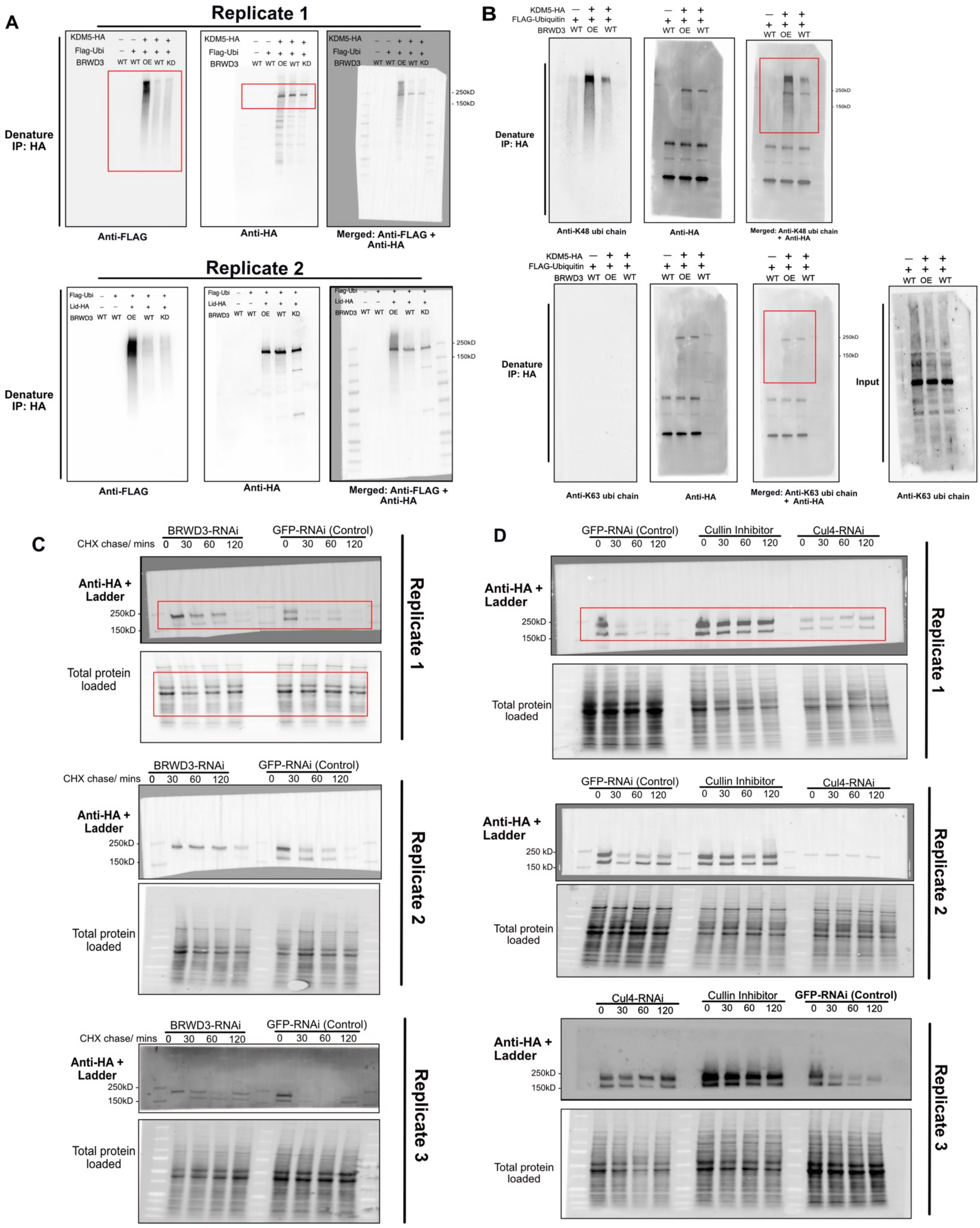
A
Replicate 1
KDM5-HA
Flag-Ubi
BRWD3 WT WT OE WT KD
250kD
150kD
Denature IP: HA
Anti-FLAG
Anti-HA
Merged: Anti-FLAG + Anti-HA
Replicate 2
Flag-Ubi
Lid-HA
BRWD3 WT WT OE WT KD
250kD
150kD
Denature IP: HA
Anti-FLAG
Anti-HA
Merged: Anti-FLAG + Anti-HA
B
KDM5-HA
FLAG-Ubiquitin
BRWD3 WT OE WT
Denature IP: HA
250kD
150kD
Anti-K48 ubi chain
Anti-HA
Merged: Anti-K48 ubi chain + Anti-HA
Denature IP: HA
Input
Anti-K63 ubi chain
Anti-HA
Merged: Anti-K63 ubi chain + Anti-HA
Anti-K63 ubi chain
C
BRWD3-RNAi
GFP-RNAi (Control)
CHX chase/ mins 0 30 60 120 0 30 60 120
Anti-HA + Ladder
250kD
150kD
Total protein loaded
Replicate 1
Replicate 2
Replicate 3
D
GFP-RNAi (Control)
Cullin Inhibitor
Cul4-RNAi
0 30 60 120
Anti-HA + Ladder
250kD
150kD
Total protein loaded
Replicate 1
Replicate 2
Replicate 3

Supplemental Figure 3. BRWD3 promotes KDM5 ubiquitination and degradation. Full Western blots and additional replicates used for Figure 3 in the main text. Red boxes indicate cropped regions. (A) Two biological replicates for the denaturing HA-immunoprecipitation followed by Western blots in Fig. 3A are shown. Blots were probed with anti-FLAG (left) and anti-HA (middle) antibodies. The transfection conditions and *BRWD3* expression conditions are indicated on the top. (B) Full Western blots for ubiquitin chain-type specific antibody probing after denaturing HA-IP in Fig. 3B. Separated channels for anti-K48-linked polyubiquitin chains (upper, left) and anti-HA (upper, middle) antibodies, and merged channels (upper, right). Similar order is shown with an anti-K63-linked polyubiquitin antibody (lower). The input samples were blotted with anti-K63-linked polyubiquitin antibody and showed signal (lower, right). (C) Full Western blots for cycloheximide assays under *BRWD3*-RNAi or *GFP*-RNAi conditions used in Fig. 3D. Two more replicates are shown below. (D) Full Western blot for cycloheximide assays under *Cul4*-RNAi, Cullin inhibitor incubation, or *GFP*-RNAi conditions used in Fig. 3F.

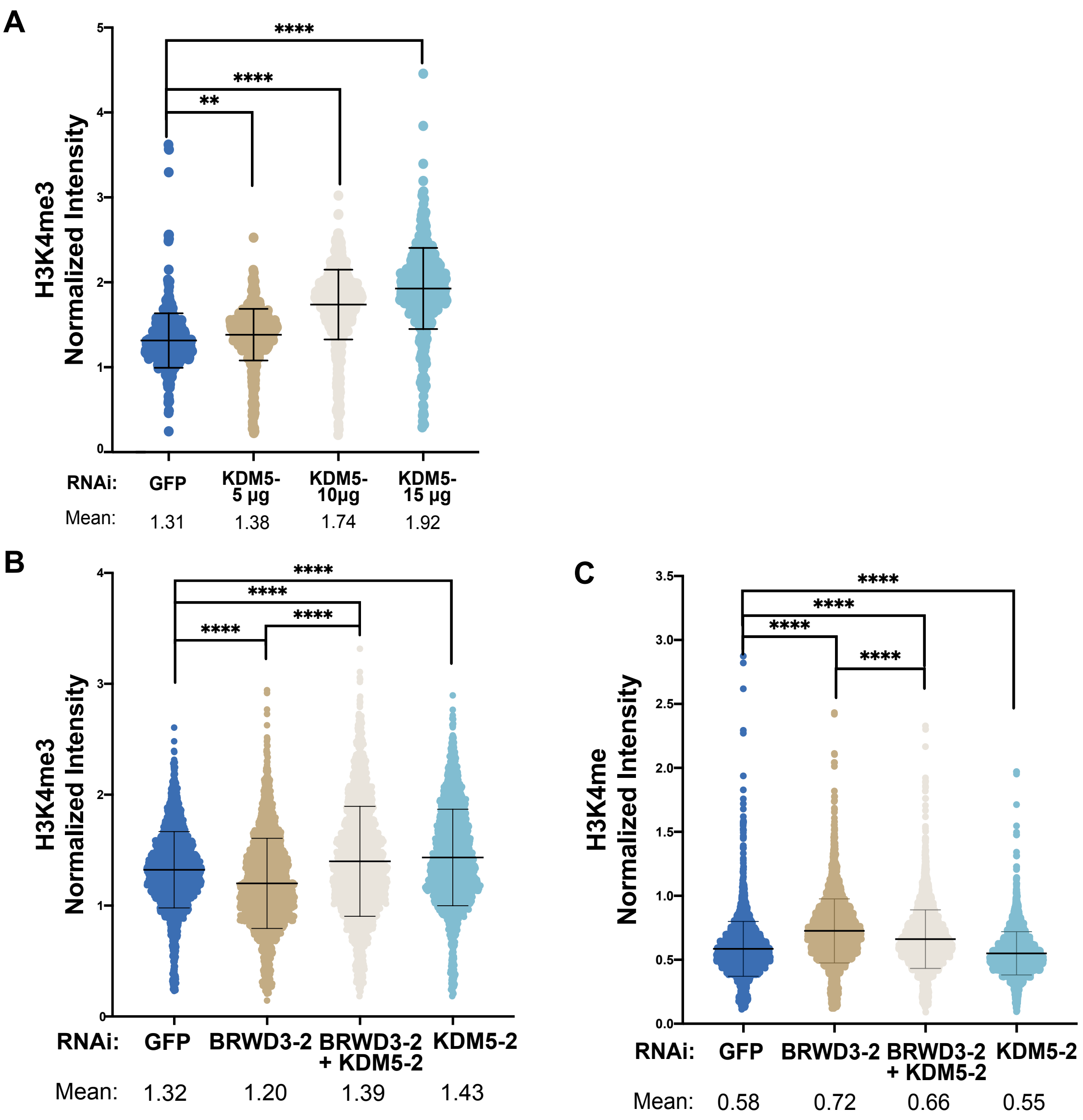

Supplemental Figure 4. Inhibition of KDM5 with an independent set of dsRNAs restores altered H3K4 methylation levels upon BRWD3 depletion. Violin plot quantitative immunofluorescence assays plot in Drosophila S2 cells using an anti-H3K4me3 antibody (A, B) or an anti-H3K4me1 antibody (C) with different dsRNA treatments. **p< 0.01, ****p < 0.0001 using ANOVA one-way analysis with Tukey's multiple comparisons test.
